# Stepwise Evolution of Immunodeficient Mouse Models: Focus on NCG Strains and Their Applications in Humanized Immune System Research

**DOI:** 10.3390/vaccines14090805

**Published:** 2026-09-13

**Authors:** Hongyan Sun, Jialu Fan, Yujing Zhang, Jun Xing, Yinlian Zhang, Cunxiang Ju, Juan Liang

**Affiliations:** GemPharmatech Co., Ltd., Nanjing 210061, China; sunhy@gempharmatech.com (H.S.);

**Keywords:** NCG, humanized mouse models, immune system reconstruction, immunotherapy evaluation

## Abstract

Immunodeficient and humanized mouse models have become essential tools for investigating human immunity, disease pathogenesis, and therapeutic evaluation. This review summarizes the stepwise evolution of these platforms, from nude and SCID mice to advanced NOD/SCID/*Il2rg^null^* strains (NCG, NSG, or NOG), and highlights how progressive removal of murine immune barriers has improved human cell engraftment and experimental utility. Major humanized immune system (HIS) approaches, including Hu-PBMC/PBL, Hu-HSC, and Hu-BLT models, are compared with respect to engraftment kinetics, immune composition, graft-versus-host disease, and suitability for distinct research applications. Particular emphasis is placed on next-generation engineered strains that enhance specific human immune compartments, including cytokine-humanized models for myeloid, dendritic cell, natural killer cell, and neutrophil reconstitution; TSLP-based models that restore secondary lymphoid organogenesis and improve adaptive immune responses; and advanced HLA-humanized models. These advances have substantially expanded the value of humanized mice in studies of autoimmune disease, cancer immunotherapy, and vaccine evaluation. Nevertheless, no single model fully reproduces the complexity of the human immune system, and major limitations remain, including incomplete stromal humanization, restricted HLA diversity, and inter-model variability. Continued refinement will be critical for improving translational relevance and predictive power in vaccine and immunology research.

## 1. Introduction

The development of immunodeficient mice has greatly advanced preclinical research by enabling the engraftment of human cells and reconstruction of human immune systems [1]. Early nude and severe combined immunodeficient (SCID) mice revealed fundamental lymphocytic deficiencies, yet their application was limited by residual immunity, immune leakage, and short experimental windows. Introduction of the non-obese diabetic (NOD) background further impaired NK-cell activity but remained constrained by spontaneous lymphoma. The milestone NOD/SCID/*Il2rg^null^* strains (NCG, NSG, and NOG), characterized by T-, B-, and NK-cell deficiency, became a gold-standard platform for xenotransplantation. Subsequent CRISPR engineering combined with human cytokine gene modification has enabled the development of humanized immune system (HIS) mice, which recapitulate human hematopoiesis and disease pathology with greater physiological relevance [2,3].

Currently, HIS mice are broadly classified into Hu-PBMC/PBL, Hu-HSC, and Hu-BLT models according to their human cell engraftment strategies [3]. However, conventional HIS models exhibit inherent limitations, including insufficient reconstitution of myeloid and dendritic cells (DCs), immature secondary lymphoid organ development, and suboptimal adaptive immune functionality [4]. Next-generation strains with human cytokine expression have been constructed to optimize human immune reconstitution [5]. This review systematically summarizes the evolutionary progress, phenotypic characteristics, limitations, and translational applications of immunodeficient and humanized mice, particularly focusing on their values in autoimmune disease research, cancer immunotherapy and vaccine development [6,7].

## 2. Evolution of Immunodeficient Mouse Models

The development of immunodeficient mouse models has evolved continuously to achieve deeper immunodeficiency and more efficient engraftment of human cells and tissues [1]. Early models represented by nude mice exhibit selective T lymphocyte deficiency due to impaired thymic development, yet their relatively weak immunodeficiency has restricted broader applications. Subsequently, SCID mice harboring the *Prkdc* mutation were established, displaying combined T and B cell deficiency but retaining functional NK cells and immune leakage. Further improvements led to the generation of NOD/SCID mice by introducing the *Prkdc* mutation into the NOD background with inherent NK cell functional defects, which improved xenograft acceptance but remained limited by residual NK activity, a high incidence of spontaneous thymic lymphoma, and shortened lifespan.

Further advancements led to the establishment of NOD/SCID mice by introducing the *Prkdc* mutation into the NOD genetic background. Although this strain exhibited enhanced xenograft engraftment capacity, it still suffered from inherent limitations, including residual NK cell activity, a high spontaneous incidence of thymic lymphoma, and a shortened lifespan. To overcome these limitations, NOD/SCID/*Il2rg^null^* mice (NCG, NSG, and NOG) were developed through targeted disruption of the IL-2 receptor gamma chain (common γ chain, *Il2rg*), enabling dramatically improved human cell engraftment without immune leakage or spontaneous thymoma formation [4]. This advanced strain is now regarded as a gold-standard highly immunodeficient platform, supporting extensive applications in immunological research and the treatment of related diseases.

### 2.1. Nude Mice

First reported in 1966, nude mice represent the earliest immunodeficient model, harboring a spontaneous Foxn1 mutation on chromosome 11 that causes thymic aplasia and selective loss of mature T lymphocytes [8]. Serum IgM is dominant with minimal IgA, enabling allogeneic tissue engraftment. Common strains, including BALB/c-nu, Swiss-nu, NC-nu, and NIH-nu, are widely used in foundational research on cancer and immune-related diseases. However, retained functional B cells and NK cells limit the efficient engraftment of human immune cells, restricting their application in advanced humanized models [2].

### 2.2. SCID Mice

Discovered in 1983, CB-17 SCID mice carry a recessive *Prkdc* mutation on chromosome 16 that impairs V(D)J recombination, blocking maturation of T and B lymphocytes and reducing serum immunoglobulin levels [9,10]. NK cells and macrophages remain functionally intact, and age-dependent immune “leakage” occurs in 2–23% of animals. To mitigate radiosensitivity, Rag1- or Rag2-knockout (*Rag^null^*) mice were developed, exhibiting stable SCID-like phenotypes without spontaneous reversion [4]. Despite these improvements, robust NK cell activity still limits efficient human cell engraftment.

### 2.3. NOD/SCID Mice

First developed in 1980, NOD mice present inherent innate immune deficiencies, such as impaired NK cell and macrophage function and defective circulating complement [11]. The NOD-specific SIRPa polymorphism further promotes human cell engraftment via inhibition of macrophage phagocytosis [4]. In 1995, the introduction of the *Prkdc* mutation generated NOD/SCID mice. These mice are deficient in mature T and B cells, with attenuated NK cell responses and impaired innate immunity, thereby supporting superior engraftment of human tumor and hematopoietic cells compared with conventional SCID mice [12]. Nevertheless, NOD/SCID mice have prominent limitations: residual NK activity, T/B cell leakage, high spontaneous thymic lymphoma incidence, a short lifespan of around 8 months, and increased radiosensitivity. Even so, they allow the development of human gut-associated lymphoid tissue (GALT) and are widely used for EBV and HIV infection models, supporting selective reconstitution of human myeloid and B cells [13,14,15].

### 2.4. NOD/SCID-Il2rg^null^ Mice

Il2rg is indispensable for signaling by IL-2, IL-4, IL-7, IL-9, IL-15, and IL-21, governing the development of T, B, and NK cells. Targeted disruption of *Il2rg* abolishes NK cell maturation and severely impairs adaptive immunity, generating fourth-generation triple-deficient mice characterized by the absence of T, B, and NK cells [16,17]. These mouse strains exhibit no immune leakage and are free from spontaneous thymoma, support unprecedented human cell engraftment, and represent the current gold standard for humanized model establishment.

Beyond the widely used NSG and NOG strains, the NCG mouse was developed through the disruption of *Prkdc* and *Il2rg* on a NOD genetic background. This is a genetically well-characterized mouse model with robust human cell engraftment and high physiological resilience (Table 1). However, the severe immunodeficient mice with the *prkdc* mutation on a NOD background inherently share certain physiological characteristics that may be viewed as limitations in specific experimental contexts. The NOD background carries a natural deletion in the hemolytic complement gene (*Hc*), resulting in C5 deficiency and impaired complement-mediated cytolytic activity, which can be a consideration for studies involving complement-dependent immune effector functions [18]. In addition, the defective DNA-dependent protein kinase catalytic subunit (DNA-PKcs) caused by the *Prkd^scid^* mutation or targeted *Prkdc* knockout leads to reduced efficiency of non-homologous end joining-mediated DNA double-strand break repair [19]. As a result, these mice exhibit increased sensitivity to ionizing radiation, which may restrict the use of irradiation-based conditioning or genotoxic treatments in certain experimental designs [20].

In addition, BRGS mice are established on a BALB/c background with expression of NOD-type *SIRPα* and display human cell engraftment efficiency comparable to that of NOD-derived immunodeficient strains [4,21]. Rag2 deficiency blocks V(D)J recombination without impairing general DNA repair pathways; BRGS mice could, therefore, tolerate higher irradiation doses than *Prkdc*-mutant strains [22]. Collectively, these immunodeficient mouse strains provide a versatile and reliable platform for the development of humanized models, supporting cutting-edge research in oncology, hematology, infectious diseases, autoimmune disorders, and metabolic diseases.

## 3. Engineered Immunodeficient Mouse Models: Cytokine Enhancement and Functional Optimization for Human Immune Reconstitution

**Figure 1:**
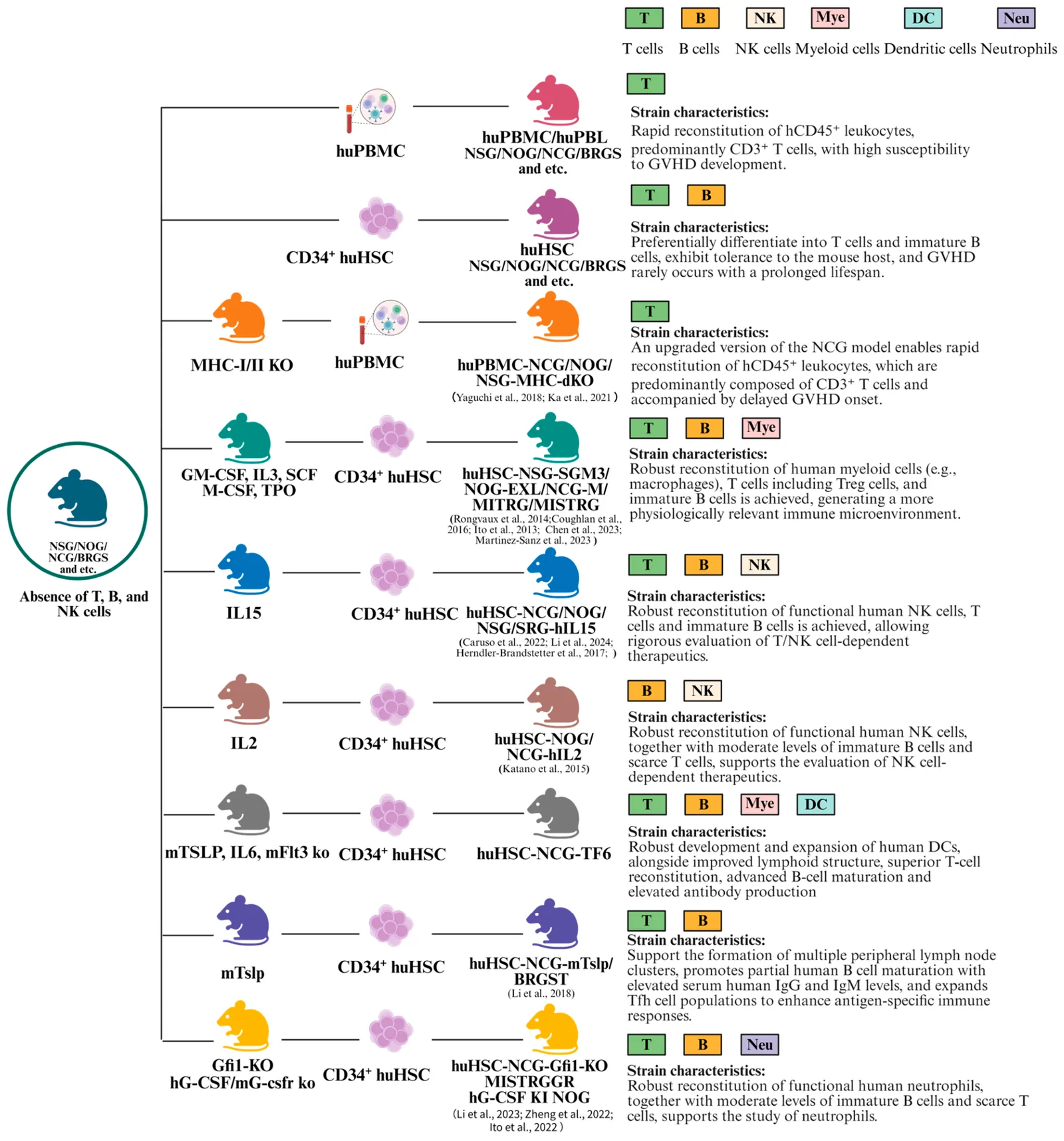
Representative advanced models for human immune reconstitution. Data sources: MHC-dKO strains [23,24]; Myeloid and DC reconstitution models including NSG-SGM3, NOG-EXL, NCG-M, MISTRG [4,25,26,27,28]; IL-15–supported NK cell models [29,30,31]; IL-2–transgenic NOG [32]; TSLP-based lymph node models [33]; G-CSF–mediated neutrophil models [34,35,36]. Created in BioRender. Nanjing, G. (2026) https://BioRender.com/xrcwaj6.

Conventional immunodeficient mouse models have limitations in human immune cell engraftment and functional simulation, restricting their preclinical application. To address these drawbacks, next-generation engineered immunodeficient mouse models have been developed, optimized with human cytokine expression, niche improvement, and secondary lymphoid structure restoration. They effectively support human immune reconstitution, facilitate accurate evaluation of immunotherapies and vaccines, and bridge the gap between basic research and clinical practice, providing reliable tools for immunology investigation and drug research. Herein, we summarize several representative advanced models of human immune reconstitution (Figure 1).

### 3.1. Myeloid-Enhanced Humanized Models

Faithful recapitulation of the human immune system requires not only functional lymphoid compartments but also robust and physiologically relevant myeloid reconstitution, including monocytes, macrophages, neutrophils, and DCs. Conventional immunodeficient strains such as NSG, NOG, and NCG support reasonable human lymphoid reconstitution but remain deficient in myeloid cell development, largely because of species-specific cytokine incompatibility: mouse-derived hematopoietic factors poorly activate receptors on human HSCs, leading to insufficient survival, differentiation, maturation, and tissue retention of human myeloid cells. To address these limitations, next-generation immunodeficient mice have been engineered to express key human cytokines that govern discrete stages of myelopoiesis and DC development, thereby more closely mimicking human hematopoiesis development.

Human myeloid development proceeds through a stepwise differentiation program tightly regulated by a cascade of hematopoietic cytokines [37,38,39]. SCF (KITLG) supports the maintenance, self-renewal, and early lineage priming of HSCs and myeloid-biased progenitors, establishing a foundational precursor pool for downstream myelopoiesis. IL-3 acts as a broad multilineage factor that stimulates the expansion and differentiation of myeloid progenitors, increasing the precursor pool for monocytes, granulocytes, DCs, and mast cells. GM-CSF (CSF2) drives myeloid progenitor commitment toward monocyte and DC lineages, enhances the maturation of monocyte-derived DCs and tissue macrophages, and elevates antigen-presenting capacity [40]. M-CSF (CSF1) selectively promotes the differentiation, survival, and tissue residency of macrophages, including liver Kupffer cells and lung alveolar macrophages [41].

To achieve robust human myeloid reconstitution, multiple cytokine-humanized immunodeficient mouse strains have been developed, categorized by genetic background and humanization strategy. Three strains harboring a NOD/SCID/*Il2rg^null^* background have been established using gene-editing technology. Among them, NSG-SGM3 (NSGS) expresses human IL-3, GM-CSF, and SCF to expand early myeloid progenitors and support the reconstitution of CD33^+^ myeloid and DCs. However, its application is compromised by severe and fatal anemia following HSC engraftment [25]. NOG-EXL mice express human IL-3 and GM-CSF to promote myeloid and eosinophil differentiation, resulting in abundant CD14^+^ monocytes that are well suited for allergy and infectious disease research [26]. NCG-M is generated via site-specific knockin at the *Rosa26* locus to ensure superior genetic stability. This strain co-expresses IL-3, GM-CSF, and SCF, supporting robust pan-myeloid reconstitution and sustained hematopoietic homeostasis. Notably, this strain supports a relatively long post-engraftment survival lifespan [27]. In contrast, BRGSF, MITRG, and MISTRG are myeloid-promoting strains derived from a *Rag2*^−/−^*Il2rg*^−/−^ background. BRGSF bears a mouse *Flt3* knockout, which robustly enhances human cell engraftment and facilitates the reconstitution of dendritic cell subsets upon exogenous administration of human FLT3L [42,43]. MITRGs were generated by site-specific knock-in of human *M-CSF*, *IL-3*, *GM-CSF*, and *TPO* genes into their respective mouse loci. This synergy between multiple humanized cytokines would enable the recapitulation of human myeloid development and function in the mouse [4]. Building on this foundation, MISTRG is maintained on the same mixed BALB/c;129 background and expresses human *M-CSF*, *IL-3*, *GM-CSF*, and *TPO*, while additionally introducing a human *SIRPα* transgene [28]. The expression of human SIRPα provides critical suppressive signaling by binding to human CD47, which is constitutively present on human cell surfaces. This specific ligand–receptor interaction effectively inhibits the phagocytic activity of murine macrophages, substantially reducing xenogeneic rejection and rendering and substantially enhancing human cell engraftment and immune reconstitution [44,45]. The above configuration enables the full recapitulation of human myeloid development and function, rendering the model highly permissive to human hematopoiesis and xenogeneic cell engraftment [4,46,47]. Nevertheless, humanized MITRG and MISTRG mice both suffer from a truncated lifespan due to severe anemia, likely attributed to irradiation injury and human macrophage-mediated clearance of murine red blood cells [48]. Collectively, these myeloid-enriched immunodeficient mouse strains enable robust reconstitution of human myeloid cells and recapitulate human innate immunity, thereby serving as promising platforms for modeling myeloid malignancies and evaluating CAR-M and CAR-T therapeutics [25,46,49].

### 3.2. DCs-Enhanced Humanized Models

Robust reconstitution of DCs is essential for reliable preclinical assessment of human vaccines. It has been reported FLT3L regulates early lymphoid-myeloid progenitors and acts as the master cytokine for the development, homeostasis, and functional maturation of both myeloid DCs (mDCs) and plasmacytoid DCs (pDCs) [43,50]. FLT3L-based models represent a critical optimization for DC reconstitution, markedly increasing the frequency of mDCs and pDCs in lymphoid organs and tissues [42]. Strains such as NSG-FLT3L and NSG-SGM3-FLT3L can achieve human DC frequencies approaching physiological human levels, with mDCs and pDCs each reaching 1–5% of hCD45^+^ cells and exhibiting strong antigen uptake, maturation, and T-cell priming capacity [51,52,53]. Among these FLT3L-optimized strains, the NCG-hIL6-mFlt3 KO-mTslp Tg mouse represents an advanced and dedicated dendritic cell research platform, commonly referred to as NCG-TF6.

NCG-TF6 incorporates multiple complementary genetic modifications to further potentiate DC function and optimize its applicability for vaccine evaluation. The human IL-6 transgene sustains stromal cell activity and preserves myeloid and lymphoid progenitor populations [33]; mouse *Flt3* knockout diminishes endogenous murine myeloid competition, thereby generating a more permissive niche for human myeloid and dendritic cell development [54]. Additionally, overexpression of mouse *Tslp* facilitates the organogenesis of secondary lymphoid organs, augments T-cell reconstitution and tissue localization, and establishes a supportive microenvironment for antigen presentation [55].

**Figure 2:**
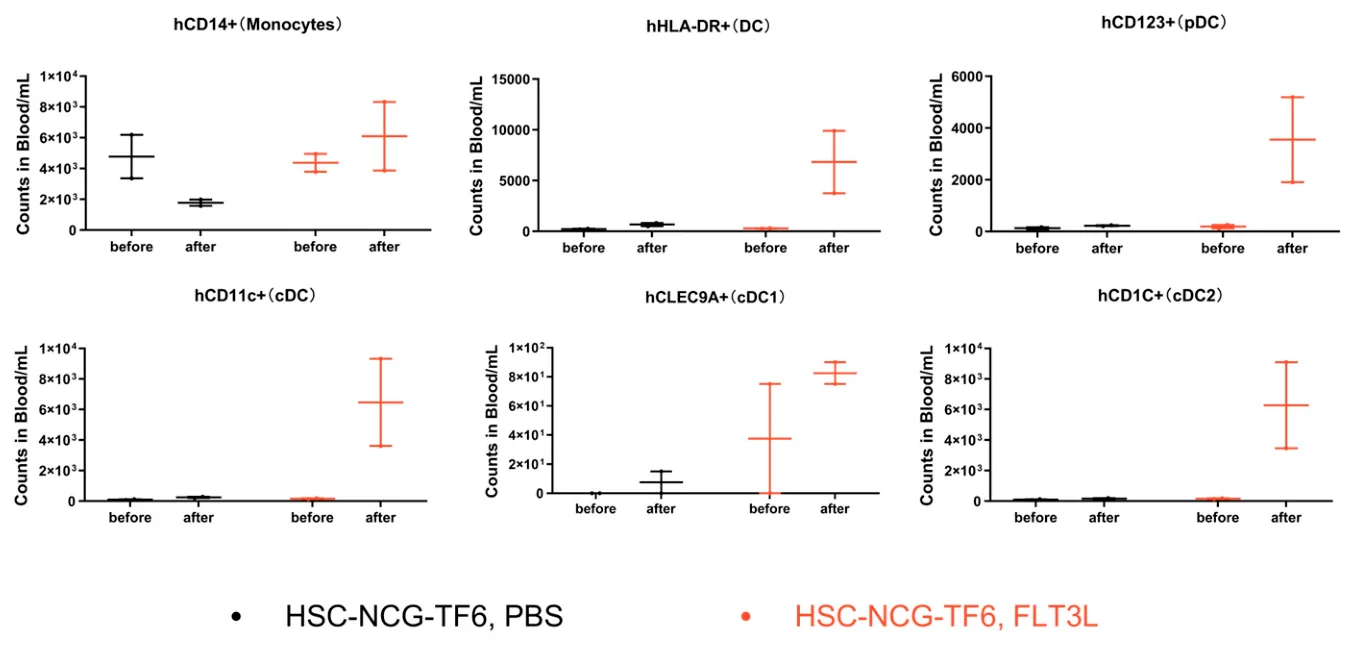
Recombinant human FLT3L treatment enhances human myeloid cell reconstitution in HSC-engrafted NCG-TF6 mice. Kinetics of human myeloid reconstitution in humanized NCG-TF6 mice upon human FLT3L protein treatment. At 18 weeks after human HSC engraftment, HSC-engrafted NCG-TF6 mice were administered 10 µg of human FLT3L protein on days 0, 2, 4, and 7. Peripheral blood was harvested for flow cytometry analysis at baseline and day 9 (*n* = 2). Data are shown as mean ± SEM.

Following human CD34^+^ HSC engraftment for 12–18 weeks combined with exogenous FLT3L administration, NCG-TF6 exhibits markedly enhanced development and expansion of human mDCs and pDCs. Its improved lymphoid structure, robust T-cell reconstitution, and functionally superior DCs collectively strengthen antigen presentation and T cell priming capacity. Furthermore, this strain facilitates advanced B-cell maturation and elevated antibody production, rendering it a promising platform for nonclinical vaccine evaluation. In a representative study, human FLT3L was administered to HSC-engrafted NCG-TF6 mice at 18 weeks post-transplantation. Compared with untreated controls, these treated mice showed markedly enhanced reconstitution of human monocytes, pDCs, and cDCs in peripheral blood (Figure 2). These findings indicate that the NCG-TF6 strain represents an optimal model for studying human DC development. Collectively, advanced humanized mouse models, including NSG-SGM3-FLT3L, MISTRG, and notably NCG-TF6, enable rigorous assessment of immunogenicity across multiple vaccine modalities. These encompass mRNA vaccines, viral vector vaccines, protein subunit vaccines, and therapeutic cancer vaccines.

### 3.3. Lymph Node-Enhanced Humanized Models

A major inherent limitation of conventional humanized mouse models such as NSG, NOG, and NCG is the impaired development of secondary lymphoid organs (SLOs). This defect stems from *Il2rg* ablation, which eliminates the common gamma chain (γc) subunit shared by multiple interleukin receptors. Lymphoid tissue inducer (LTi) cells—essential for embryonic lymph node (LN) organogenesis—rely critically on γc-dependent IL-7 signaling [56,57,58]. Accordingly, *Il2rg* deficiency results in a significant loss of lymph nodes and severely disorganized splenic structure, thereby markedly restricting the ability of these models to mount robust antigen-specific immune responses after HSC engraftment.

**Figure 3:**
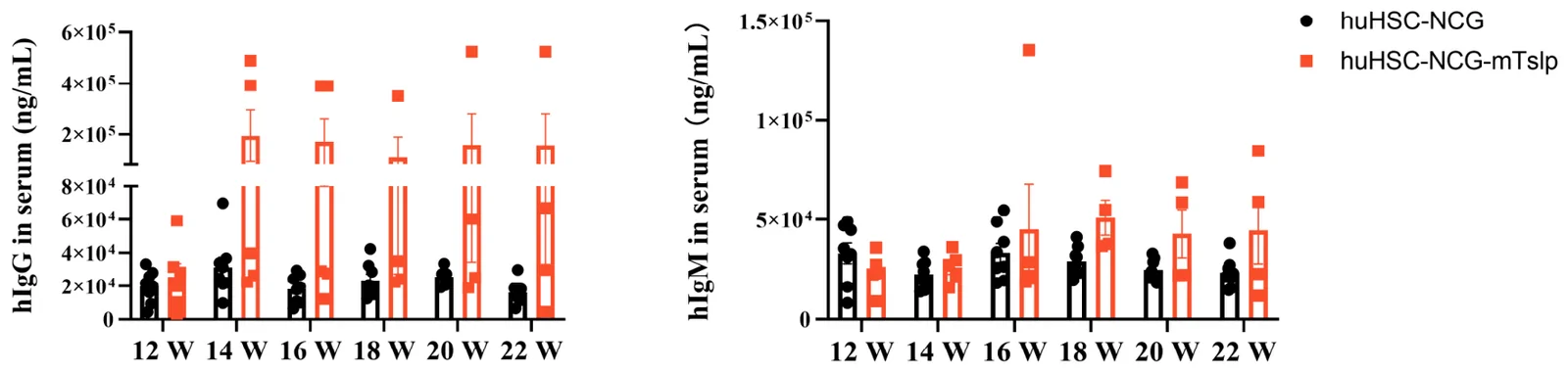
Detection of human IgG and IgM in the serum of huHSC-NCG-mTslp mice. Serum samples were collected from huHSC-NCG and huHSC-NCG-mTslp mice at 12–24 weeks post-immune reconstitution and subjected to ELISA detection. Data are shown as the mean ± SEM.

**Figure 4:**
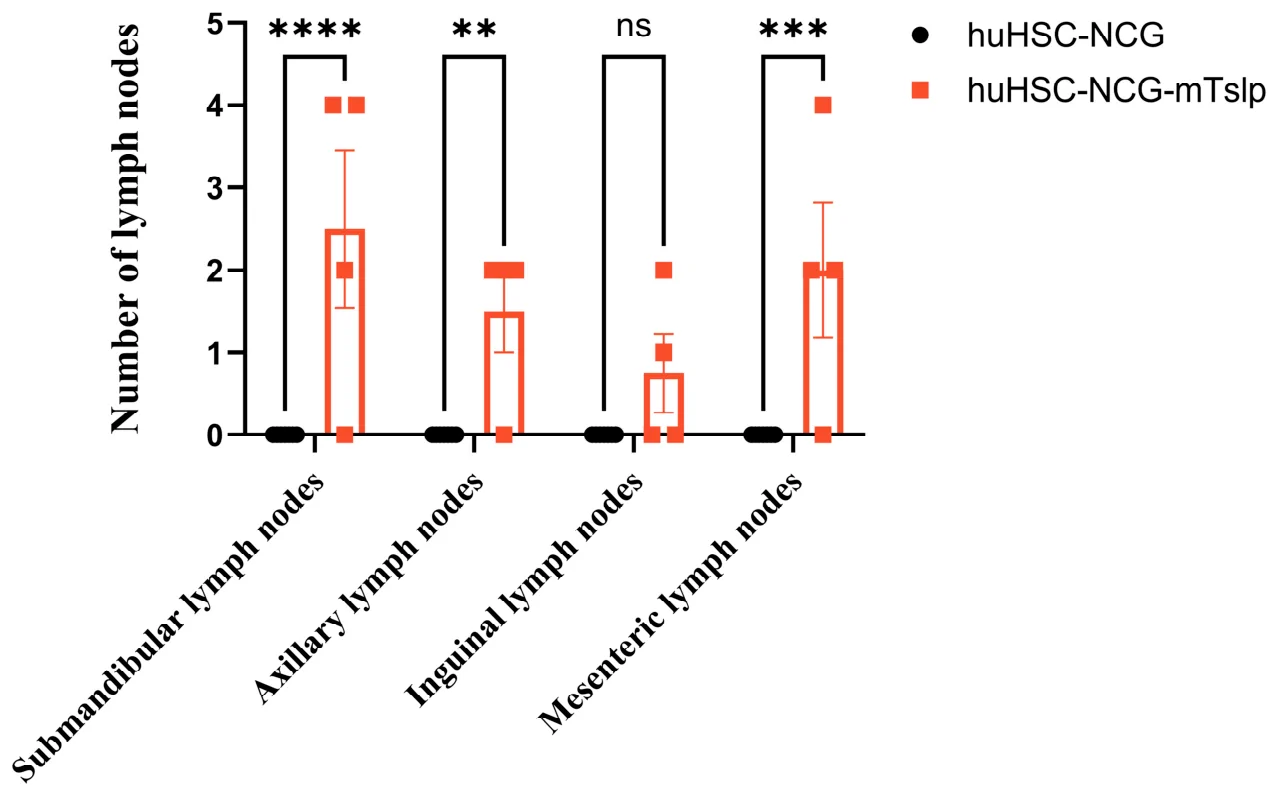
Lymph node profiling of huHSC-NCG-mTslp mice at 24 weeks post immune reconstitution. Lymph node tissues were isolated from huHSC-NCG (*n* = 7) and huHSC-NCG-mTslp (*n* = 4) at 24 weeks after immune reconstitution. Data are presented as mean ± SEM. Statistical analyses were performed using GraphPad Prism 10 with Two-way ANOVA. A *p*-value of less than 0.05 was considered statistically significant. Significance in the figures is indicated as follows: ** *p* < 0.01, *** *p* < 0.001, **** *p* < 0.0001; ns, not significant (*p* ≥ 0.05).

To circumvent this bottleneck, thymic stromal lymphopoietin (TSLP) has emerged as a pivotal regulator for rescuing LN development. Despite sharing structural and functional homology with IL-7, TSLP initiates signaling through the IL-7Rα/TSLPR heterodimer in a γc-independent manner. This alternative signaling route allows TSLP to evade γc deficiency, thereby sustaining LTi cell development and facilitating the assembly of functional secondary lymphoid structures [58,59,60]. Notably, transgenic murine Tslp expression in BRGS mice was first shown to drive the full maturation of compartmentalized LNs reconstituted with human B and T cells. This intervention also results in thymic enlargement, augmented mature B cell populations, and expanded IL-21-secreting follicular helper T (Tfh) cells, collectively boosting antigen-specific immune reactivity [33]. Building upon this foundation, we generated a series of *Tslp*-engineered mouse models on the NCG background to accommodate diverse research requirements. In the huHSC-NCG-mTslp model, elevated serum levels of human IgG and IgM indicate partial B cell maturation (Figure 3). Furthermore, the formation of multiple lymph node clusters, including submandibular, axillary, inguinal, and mesenteric lymph nodes, supports the efficient reconstitution of secondary lymphoid structures (Figure 4).

In addition to the TSLP modification strategy for rescuing defective secondary lymphoid organs, cytokine humanization serves as a critical approach to optimize lymphoid structure and overall immune function. RG SKI IL-6 mice carry knock-in human *SIRPα* together with physiologically expressed human *IL-6*. After antigen immunization, this strain generates abundant antigen-specific IgG memory B cells with diversified antibody repertoires and elevated somatic hypermutation, making it suitable for humoral immunity research [61]. Nevertheless, unlike TSLP-engineered BRGS/NCG strains, RG SKI IL-6 mice cannot form intact splenic germinal centers and organized lymphoid compartments [61]. Beyond improved T and B cell maturation shared with RG SKI IL-6 mice, MISTRG and MISTRG6 mice carrying *SIRPα* and *IL-6* genes can develop human monocyte subsets and fully functional NK cells, which are almost undetectable in conventional humanized mouse strains [4,62,63]. In addition, driven by physiological human IL-6 signaling, MISTRG6 enables markedly longer retention of human tissue-resident T cells relative to conventional MISTRG mice [64]. Benefiting from human myeloid and lymphoid compartment reconstitution, MISTRG6 exhibits superior translational value for inflammatory and infectious disease research [64,65,66].

Notably, in addition to introducing cytokines targeting lymph node organogenesis, exogenous estrogen treatment has been reported as another approach to facilitate the reconstitution of lymphoid lineages and lymph node tissues [67]. The THX humanized mouse model is generated by intracardiac transplantation of human cord blood-derived CD34^+^ HSC into neonatal NBSGW and NSGW41 mice, with continuous oral 17β-estradiol supplementation initiated at 14–18 weeks after immune reconstitution. Sustained estradiol treatment augments human lymphoid and myeloid engraftment, thymic epithelial maturation, and secondary lymphoid tissue formation; the model supports full somatic hypermutation and antibody class-switch recombination, induces potent neutralizing antibodies post-vaccination, and recapitulates lupus-like autoimmunity, thus acting as a flexible preclinical tool for vaccine evaluation and autoimmune research [67].

### 3.4. Advanced NK Cell Humanized Models

NK cells have emerged as a promising platform for cancer immunotherapy owing to their potent anti-tumor activity, low graft-versus-host disease (GVHD) risk, and capacity to potentiate the efficacy of antibodies via antibody-dependent cellular cytotoxicity (ADCC) [68,69]. Nevertheless, the preclinical advancement of NK cell-based therapeutics is constrained by insufficient engraftment and impaired in vivo persistence of human NK cells in conventional immunodeficient mice, which fail to produce human cytokines indispensable for NK cell development and survival [70]. To overcome this limitation, next-generation humanized mouse models have been genetically engineered to express human cytokines. Of these, strains harboring human IL-15 or IL-2 are of particular value. The hIL15 and hIL2 humanized mouse models therefore serve as robust platforms for dissecting human NK cell biology and preclinically assessing NK cell-based immunotherapies.

IL-15 is a non-redundant cytokine indispensable for the development, survival, and activation of both T cells and NK cells [71]. Two mouse strains, designated NOG-hIL15 and NCG-hIL15, with a NOD/SCID/*Il2rg^null^* background, were established by utilizing gene editing to drive IL-15 expression [29,30]. These immunodeficient mouse models engineered to express IL-15 have been widely utilized in preclinical studies of ADCC-associated research and CAR-NK cell therapy, as well as innovative drugs targeting both T and NK cells [29,30,72]. In a representative study, NCG-hIL15 mice engrafted with human CD34^+^HSCs supported robust reconstitution of functional human T and NK cells. These mice were further implanted with Raji B-cell lymphoma cells to establish a xenograft model and then treated with the anti-CD20 antibody rituximab. Rituximab treatment induced dose-dependent suppression of tumor growth, and therapeutic efficacy was positively correlated with the abundance of tumor-infiltrating human NK cells [73]. In addition, the NCG-hIL15 model has been adopted for autoimmune disease research, as exemplified by a patient-derived PBMC-induced type 2 diabetes model, which reveals the pivotal involvement of immune cells in metabolic inflammation [74]. Furthermore, SRG-15 mice are generated by knock-in of human *SIRPA* and *IL-15* genes on a *Rag2^−/−^ Il2rg^−/−^* immunodeficient background with a 129 × BALB/c genetic background, which differs from the NOD-derived backbone of NOG-hIL15 and NCG-hIL15 strains. Functionally, human NK cells developed in SRG-15 possess intrinsic cytotoxic capacity without prior exogenous pre-activation [31]. Consistent with the anti-tumor phenotype observed in NCG-hIL15 lymphoma models, rituximab monotherapy triggered robust ADCC responses and prominent tumor regression only in SRG-15 humanized mice bearing the Raji B-cell lymphoma xenograft model, which directly validates its superiority for evaluating antibody-mediated NK-dependent anti-tumor immunotherapy [31,75]. Collectively, these IL-15 humanized mouse models enable reliable nonclinical evaluation of immunotherapies and autoimmune pathologies that concurrently engage the effector functions of T and NK cells.

**Figure 5:**
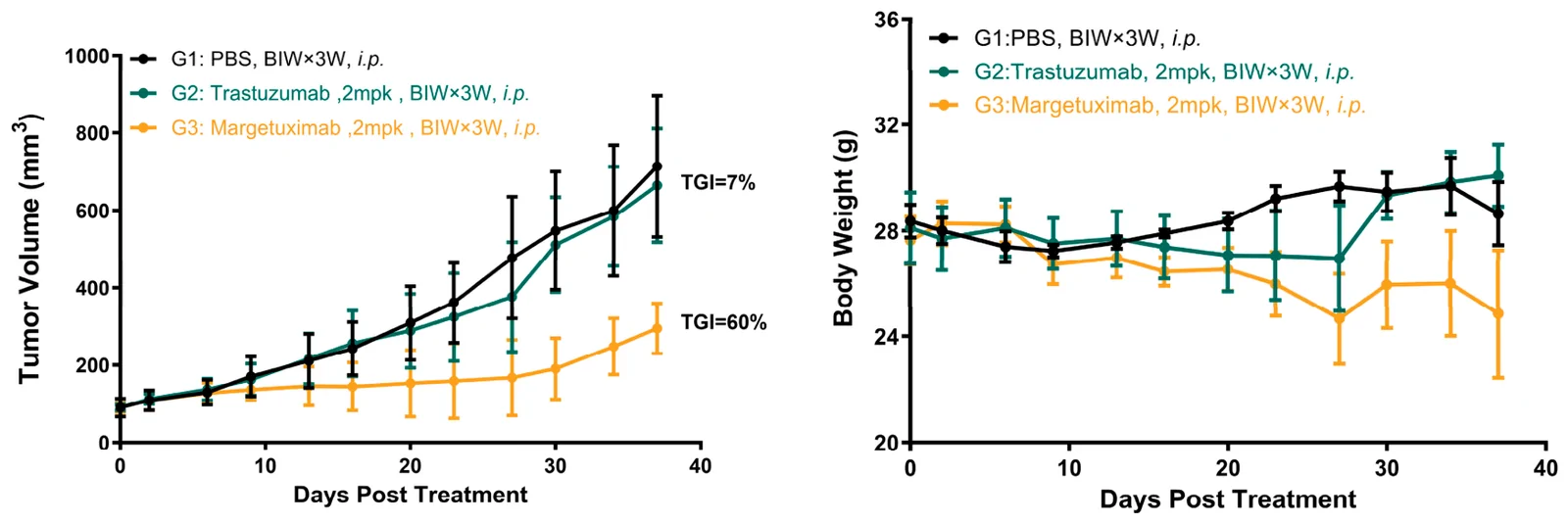
Evaluation of ADCC therapeutic agents in JIMT1 tumor-bearing huHSC-NCG-hIL2 mice. JIMT1 tumor cells were inoculated subcutaneously into female huHSC-NCG-hIL2 mice (*n* = 5 per group). When tumor volume reached 80–100 mm^3^, treatment with trastuzumab and margetuximab was initiated at a dose of 2 mg/kg per mouse via intravenous injection, administered twice weekly for 3 consecutive weeks (6 doses in total). Body weight and tumor volume were monitored twice weekly. Data are shown as mean ± SEM.

IL2 is an indispensable cytokine critical for the proliferation, survival, and activation of NK cells as well as regulatory T cells [32,76,77]. Notably, humanized IL-2 mouse strains such as NCG-hIL2 and NOG-hIL-2 Tg consistently exhibit robust reconstitution of functional human NK cells following engraftment with human CD34^+^ HSCs, accompanied by only minimal T cell development. This phenotype is largely attributed to the essential function of human IL-2 signaling in driving NK cell differentiation and maturation after HSC transplantation [32]. This unique phenotypic feature differentiates IL-2 humanized models, offering prominent advantages for studying antibody-dependent cellular cytotoxicity (ADCC) mechanisms in the absence of T cell-mediated interference. In the huHSC-NCG-hIL2 humanized mouse model engrafted with HER2-positive, trastuzumab-resistant JIMT-1 breast cancer cells, margetuximab with enhanced ADCC activity exhibited stronger potent tumor growth inhibition than conventional trastuzumab with basal ADCC function. These results confirm that this model can reliably distinguish the differences in ADCC potency (Figure 5). However, this model still contains resident mouse macrophages that could respond to margetuximab in an Fcγ receptor-dependent manner, potentially confounding the interpretation. This limitation could be addressed by further knockout of Fcγ receptors to eliminate mouse macrophage interference.

### 3.5. A Specialized Model for Human Neutrophil Reconstitution

Neutrophils, the most abundant circulating leukocytes in humans, play multifaceted and context-dependent roles in cancer biology. While traditionally recognized as first-line defenders against infection, tumor-infiltrating neutrophils (TINs) have been increasingly appreciated for their immunosuppressive and tumor-promoting functions, a phenotype closely associated with the polymorphonuclear myeloid-derived suppressor cell (PMN-MDSC) population. Despite their clinical relevance, studying human neutrophils in vivo has been notoriously challenging due to their short half-life and the poor engraftment of these cells in conventional humanized mouse models [78].

Granulocyte colony-stimulating factor (G-CSF) is a master regulator of granulopoiesis, signaling through its cognate receptor G-CSFR to drive the proliferation, differentiation, and survival of neutrophil precursors [79,80]. However, human G-CSF exhibits limited signaling activity via mouse G-CSFR, which accounts for defective human neutrophil reconstitution in standard humanized mouse models. Consequently, CD34^+^HSC-engrafted NSG, NCG, and other classic immunodeficient strains display profoundly impaired human myeloid engraftment, with neutrophil differentiation particularly blunted [63]. To overcome this limitation, several strategies have been developed to provide human G-CSF signaling. The MISTRGGR humanized mouse model humanizes *G-CSF* and knocks out murine *G-CSFR*, enabling robust reconstitution of mature and functional human neutrophils in circulation and tissues [35]. Similarly, the hG-CSF knock-in (KI) NOG mouse, which expresses human G-CSF under the endogenous murine G-CSF promoter, has also been shown to support significantly improved human neutrophil engraftment and maturation following HSC transplantation [36]. Moreover, one alternative strategy is to create a permissive niche for human myeloid progenitor engraftment through genetic ablation of endogenous murine myeloid differentiation competition. The transcription factor growth factor independence 1 (*Gfi1*) serves a critical, non-redundant function in murine granulopoiesis [81]. Gfi1 is indispensable for the differentiation of common myeloid progenitors (CMPs) and granulocyte-monocyte progenitors (GMPs) into mature neutrophils. *Gfi1* knockout mice exhibit altered proportions of CMPs and GMPs and are completely devoid of mature neutrophils, with a concomitant accumulation of immature monocytic cells [82,83]. This profound impairment in the endogenous murine neutrophil differentiation pathway generates a vacant myeloid niche, which can be preferentially occupied by engrafted human hematopoietic progenitors. Leveraging this biological mechanism, we generated the NCG-Gfi1-KO mouse strain. This model has proven instrumental in delineating the stage-specific functions of human neutrophils [34,78]. NCG-Gfi1^−/−^ HIS mice could support robust reconstitution of human neutrophils across multiple tissues, including bone marrow, spleen, peripheral blood, and the tumor microenvironment. Human neutrophils derived in NCG-Gfi1^−/−^ HIS mice exhibit hallmark effector functions, including chemotactic migration toward CXCL8 in vitro, formation of neutrophil extracellular traps (NETs) upon PMA stimulation, and potent immunosuppressive activity, as evidenced by their ability to suppress T-cell proliferation in co-culture assays—mirroring the functional phenotype of primary human bone marrow neutrophils. The model also recapitulated the dynamic redistribution observed in cancer patients: a significant increase of human neutrophils in the peripheral blood and a concurrent decrease of banded and segmented (BD&SC) neutrophils were observed when tumor-bearing, indicating active mobilization from the bone marrow reservoir to the periphery [34]. The huHSC-NCG-Gfi1-KO model represents a substantial advance in human neutrophil reconstitution, providing the research community with an unprecedented platform to explore the complex, clinically relevant roles of human neutrophils.

### 3.6. HLA Humanized Models

In humans, the major histocompatibility complex (MHC) molecules are known as human leukocyte antigen (HLA) molecules, which serve as the core mediators of antigen presentation in adaptive immune responses [84,85]. T cell recognition of antigens is governed by the principle of MHC restriction, with CD4 and CD8 co-receptors that determine the functional specificity of T cell subsets. CD4^+^ T cells specifically bind to HLA class II molecules (e.g., HLA-DR, -DQ, -DP) on antigen-presenting cells (APCs) via their surface CD4 molecules. This interaction not only stabilizes the T cell receptor (TCR) binding to the peptide-HLA complex but also recruits Lck kinase to initiate downstream signaling, leading to helper T cell activation and cytokine secretion. Conversely, CD8^+^ T cells recognize HLA class I molecules (e.g., HLA-A, -B, -C) expressed on nearly all nucleated cells via CD8, enabling them to monitor and eliminate virus-infected or malignant cells [86,87,88]. Furthermore, during thymic development, HLA molecules shape the self-tolerance and diversity of the peripheral T cell repertoire through positive and negative selection, ensuring that the immune system maintains homeostasis while accurately recognizing non-self antigens [89,90]. Therefore, conventional Hu-HSC models exhibit limited T-cell maturation and impaired antigen-specific immune responses due to the lack of human HLA molecules [91]. While the BLT model supports HLA-restricted T-cell development by providing an autologous human thymic microenvironment, the reliance on human fetal thymus and liver tissue raises significant ethical concerns and limits broad applicability. To address this limitation, transgenic mouse models expressing human HLA molecules have been developed.

HLA transgenic mice were originally generated on immunocompetent backgrounds. For instance, HLA-A*02:01 transgenic mice on a wild-type murine MHC-sufficient background are capable of eliciting HLA-A2-restricted CD8^+^ T-cell responses against human-derived viral epitopes, providing early proof that human HLA molecules can function in the mouse thymic environment to educate functional T cells [92]. Among the diverse monochain constructs tested, the HHD design—which combines human β2-microglobulin and human α1α2 domains with the mouse H-2Db α3, transmembrane, and cytoplasmic domains—was identified as the most effective structure for restoring a peripheral CD8^+^ T cell repertoire educated on the transgenic HLA molecule [93]. When these HLA-humanized genes are introduced into severely immunodeficient strains such as NSG mice, they elicit robust HLA-A-restricted CD8^+^ T-cell responses following human HSC engraftment [94,95]. In the NSG-HLA-A2/HHD model, human CD4^+^ and CD8^+^ T cells exhibit enhanced functionality, with competent expression of cytotoxic molecules and cytokine secretion in vivo. Most importantly, these humanized mice have demonstrated functional HLA-restricted CTL responses in an in vivo Epstein–Barr virus (EBV) infection model, thereby verifying their potential to establish potent antiviral cellular immunity [94]. Distinct from HLA class I transgenic mice that predominantly potentiate CD8^+^ T cell immunity, HLA class II humanized models have been shown to effectively improve human B-cell function. For instance, NOG mice transgenic for HLA-DRA and HLA-DRB1*04:05 specifically express HLA-DR4 in MHC class II-positive cells and facilitate efficient engraftment of HLA-matched human HSCs [96]. Compared with HLA-mismatched control groups, these mice display distinct regulation of T cell homeostasis, and exogenous antigen immunization effectively induces antibody class switching.

Given that HLA class I and class II molecules are both indispensable for adaptive immunity, with the former mediating antigen presentation to CD8^+^ T cells and the latter to CD4^+^ T cells, they collectively coordinate cellular cytotoxicity, T helper functions, and humoral immune responses. Accordingly, transgenic mouse models carrying only a single type of HLA molecule are intrinsically incapable of fully recapitulating the integrated human immune regulation. To better reconstitute a complete and functional adaptive immune system, several models co-expressing HLA class I and class II have been generated. The most widely studied combinations pair HLA-A*02:01 with common HLA-DR alleles such as HLA-DRB1*01:01 (DR1), HLA-DRB1*04:01 (DR4), or HLA-DRB1*15:01 (DR2), as these are among the most frequent class I and class II alleles in human populations [97]. Typical dual HLA humanized strain NSG-A2/DR1 could elicit potent HLA-A2-restricted CD8^+^ T-cell responses and secrete neutralizing antibodies following viral challenge [98]. Another well-established model, DRAGA mice, which carry HLA-A2/DR4 on an NRG background, exhibit improved T and B cell functions and have been applied to studies of influenza, malaria, and other pathogens [99,100,101,102]. Additionally, BRGS-A2/DR2 mice are capable of generating robust antigen-specific T-cell immunity and IgG responses upon vaccination with a modified vaccinia virus Ankara vector encoding an HIV-derived polyprotein [103]. Collectively, these dual-HLA I/II humanized mice overcome the individual shortcomings of single-class transgenics by simultaneously supporting HLA-restricted CD8^+^ T cell cytotoxicity and CD4^+^ T-cell-dependent antibody production, thus providing a superior platform for recapitulating human adaptive immunity and evaluating vaccine and immunotherapeutic strategies.

## 4. Classification and Characteristics of Humanized Immune System (HIS) Mouse Models

HIS mice serve as vital preclinical platforms that enable in vivo investigation of human immunity, infectious diseases, and cancer [1]. Based on the strategies for human immune system reconstitution, these models are categorized into three major types: Hu-PBMC/PBL, Hu-HSC, and Hu-BLT models [3]. Each model differs substantially in engraftment procedure, immune cellular composition, reconstitution stability, and experimental suitability, thereby accommodating distinct research demands across immunology, virology, and oncology.

### 4.1. Hu-PBMC/PBL Mouse Model

The Hu-PBMC/PBL model is established via intravenous tail vein or intraperitoneal injection of human peripheral blood mononuclear cells (PBMCs) or peripheral blood lymphocytes (PBLs) into severely immunodeficient mouse strains, such as NCG, NSG, and NOG. Within two weeks post engraftment, human CD45^+^ cells typically account for 20–50% of cells in peripheral blood and the spleen, with reconstitution predominantly dominated by human CD3^+^ T cells and displaying pronounced donor-dependent variability [1].

However, this model is constrained by GVHD driven by MHC mismatch, which typically develops at 4–6 weeks post-engraftment [104]. GVHD triggers lethal immune rejection of murine tissues, markedly shortening the experimental time window, inducing nonspecific T cell expansion, and ultimately confounding the evaluation of immunotherapy efficacy. To address this limitation, MHC-I/II double-knockout (dKO) strains have been engineered via ablation of genes critical for MHC expression, including B2m, H2-K1, H2-D1, and H2-Ab1. This genetic modification prevents human T cell recognition of murine antigens and substantially alleviates GVHD progression [104]. Representative widely adopted platforms include NCG-MHC-dKO, NSG-MHC-I/II-dKO, and NOG-dKO mice; these models operate via a conserved underlying mechanism yet exhibit subtle differences in reconstitution performance [23,24].

### 4.2. Hu-HSC Mouse Model

The Hu-HSC model involves transplanting human CD34^+^ HSCs into sublethally irradiated immunodeficient mice via intrahepatic injection in neonates or tail vein injection in adults [5,105]. Early HSC engraftment in NOD/SCID mice led to poor T-cell development effinciency, while updated models based on NCG, NSG, or NOG mice enable robust and highly reproducible human immune reconstitution. Human HSCs preferentially differentiate into T cells and immature B cells in this system, with only minimal generation of NK cells, myeloid cells, and other subordinate lineages, and human CD45^+^ cells account for 25–60% of peripheral blood cells at 12 weeks after engraftment. Since immune cells are newly generated from engrafted human HSCs, they exhibit tolerance to the mouse host, and GVHD rarely occurs in this model [5,106]. Therefore, the model maintains a stable reconstitution period of over 20 weeks, which allows long-term investigations into hematopoiesis and chronic infections. While conventional Hu-HSC modeling relies on sublethal irradiation of recipient mice to deplete endogenous HSCs and create a niche for donor cell engraftment [3]. Irradiation, however, can increase mouse mortality and interfere with immune reconstitution, with the NOD/ShiLtJ background being particularly radiosensitive [107,108]. To circumvent these toxicities, immunodeficient mouse strains carrying Kit (c-Kit/CD117) receptor W41 mutations have been developed. This mutation reduces endogenous murine HSC competitiveness, creating an “open” bone marrow niche that enables robust human HSC engraftment without irradiation [109,110] Among these, NSGW41 (NSG background, Kit^W41/W41^), NBSGW (NSG background, Kit^W-41J^), and NCG-X (NCG background, c-Kit W41 mutation) are representative irradiation-free platforms [110,111,112]. In addition to permitting HSC engraftment without irradiation conditioning, these strains facilitate early human erythroid development in the bone marrow [113]. Kit-mutant mouse strains, therefore, constitute an indispensable preclinical platform for evaluating gene and cell therapies targeting β-thalassemia and other erythroid disorders [112,114].

However, this model presents limited T-cell maturation and impaired antigen-specific immune responses owing to the absence of human HLA molecules [91]. In conventional Hu-HSC models (i.e., NCG/NSG/NOG engrafted with human CD34^+^ hematopoietic stem cells), human T-cell precursors migrate to the murine thymus and undergo positive and negative selection on mouse thymic epithelial cells. Positive selection is mediated by mouse MHC molecules (H-2) rather than human HLA, resulting in a human T-cell repertoire that is restricted by murine MHC. Consequently, these T cells recognize antigens presented by mouse antigen-presenting cells (APCs) but respond poorly to antigens presented by human APCs in an HLA-dependent manner, severely limiting the ability to study human HLA-restricted T-cell responses [115,116]. Furthermore, negative selection on a murine thymic stroma may incompletely purge autoreactive T cells that would recognize human-specific self-antigens, leading to potential autoimmunity, while at the same time failing to establish robust tolerance to human-specific tissue antigens [117]. The peripheral TCR repertoire that develops under these conditions is often oligoclonal and skewed, with reduced diversity compared to the human peripheral repertoire and an over-representation of certain Vβ families; repertoire diversity may further contract over time due to homeostatic proliferation in a lymphopenic environment [115].

### 4.3. Hu-BLT Mouse Model

To overcome the murine MHC-restricted T-cell education and the consequent lack of HLA-dependent antigen-specific immune responses that characterize standard Hu-HSC models, the Hu-BLT model was designed to provide an autologous human thymic microenvironment [4]. The Hu-BLT model evolved from the earlier SCID-hu model by combining surgical engraftment of human fetal thymus and liver tissue under the mouse kidney capsule with intravenous transplantation of autologous CD34^+^ HSCs [4]. Current optimized BLT models adopt NCG, NSG, or NOG mice to enhance tissue engraftment efficiency and prolong model lifespan. While GVHD tends to develop at approximately 20 weeks post-engraftment, triple-knockout TKO-BLT mice with *Rag2*, *Il2rg*, and *Cd47* gene deficiencies remain free of GVHD until 45 weeks of age [118]. A core merit of BLT mice is their ability to form a humanized thymic stromal microenvironment [119]. Implanted human fetal thymic tissue develops into a functional thymic organoid composed of human stromal and epithelial cells. This human niche supports HLA-restricted T cell differentiation, establishes central immune tolerance, and generates a broad, functionally competent TCR repertoire [120,121].Consequently, the model enables multilineage reconstitution of human immune cells, including T cells, B cells, NK cells, DCs, and myeloid cells, together with the formation of mucosal tissues and secondary lymphoid organs [122,123]. BLT mice mount detectable antigen-specific IgM and IgG antibody responses, indicating a substantial degree of human adaptive immune functionality, though the overall architecture and regulation of these responses do not yet fully recapitulate those of the intact human immune system [124]. Accordingly, BLT mice have been validated for use in HIV infection studies and are capable of generating DENV-specific antibody responses upon immunization [125,126]. The presence of HLA-restricted T-cell responses makes the BLT model particularly valuable for vaccine evaluation, as it permits the assessment of vaccine-elicited cellular immunity and the identification of immunodominant epitopes presented by human HLA molecules. Nevertheless, the reliance on sophisticated surgical manipulation and human fetal tissue resources restricts the widespread popularization and application of this model.

## 5. Humanized Mouse Models of Autoimmune Diseases Through Immune Reconstitution

In recent years, immunodeficient mice reconstituted with a human immune system have enabled partial reconstruction of human immunity and the simulation of the in vivo human microenvironment [127]. Autoimmune diseases induced or spontaneously developed in such reconstituted systems represent an integration of “immune reconstitution” and “autoimmune disease”, preserving key features of the human immune system while reproducing the pathological processes of human autoimmunity.

### 5.1. Systemic Lupus Erythematosus (SLE) Models

SLE is a multisystem autoimmune disorder characterized by antinuclear antibody production, immune complex deposition, and chronic inflammation in multiple organs [128]. Humanized SLE models are mainly constructed through two strategies: transplantation of SLE patient-derived PBMCs/PBLs into immunodeficient mice and human HSC engraftment followed by chemical induction. Hu-PBMC models are most widely used for rapid humanization [129] and have been applied to evaluate the efficacy of diverse therapeutics targeting autoantibodies, pathogenic B cells, and pro-inflammatory factors [130,131]. After PBMC engraftment, NCG mice faithfully recapitulate B-cell dynamics and autoantibody progression in human SLE and respond stably to TCE and CAR-T therapy, making them suitable for preclinical drug assessment [132]. Nevertheless, this model shows insufficient reconstitution of B, NK, and myeloid cells; low uniformity, and short survival. Newly established models using pre-treated ddY mice with SLE HSC transplantation effectively mimic autoantibody profiles and hematopoietic abnormalities, yet lack pathological tissue evaluation and suffer limited donor samples [133].

HSC-induced SLE models exhibit higher stability. Neonatal NSG mice with sublethal irradiation intrahepatically transplanted with human CD34^+^ HSCs and pristane induction present comprehensive immune reconstitution and typical organ lesions in the kidney and lung [134]. NCG-M mice facilitate myeloid and lymphoid development, based on the constructed TLR7 agonist-induced humanized SLE model [135,136]. Collectively, current humanized SLE models still face obstacles, including inefficient B-cell reconstitution, low survival, and absence of standardized commercial models.

### 5.2. Rheumatoid Arthritis (RA) Models

RA is a chronic inflammatory joint disease featured by synovitis, bone erosion, and joint deformity [137]. NSG mice engrafted with RA patient PBMCs combined with anti-type II collagen antibody and LPS induction replicate key pathological features of RA and are validated for drug screening but only reflect acute inflammation with obvious donor variability [138]. NCG-M mice reconstituted with human HSCs and challenged by complete Freund’s adjuvant (CFA) closely mimic chronic human RA lesions with immune cell infiltration, and adalimumab treatment effectively alleviates disease, serving as a robust preclinical platform. Other models including EBV-infected NOG mice and B/memory T cell-transplanted immunodeficient mice support mechanistic and pharmacological studies of RA [139].

### 5.3. Inflammatory Bowel Disease (IBD) Models

IBD, consisting of ulcerative colitis (UC) and Crohn’s disease (CD), presents chronic intestinal inflammation. Conventional models fail to replicate patient heterogeneity and fibrosis, leading to high clinical trial failure rates. NSG mice transplanted with CD patient PBMCs spontaneously develop intestinal remodeling and fibrosis without extra stimulation, while UC PBMC-engrafted mice require ethanol induction to show severe inflammation with mild fibrosis, and model histology correlates with donor clinical scores [140]. These models enable direct evaluation of human-targeted drugs but are restricted by large donor differences and technical complexity.

### 5.4. Asthma Models

Asthma is characterized by airway hyperresponsiveness and eosinophilia [141]. Conventional mice cannot support human mast cell and eosinophil differentiation. NOG mice expressing human IL-3/GM-CSF after HSC engraftment and IL-33 induction reproduce typical asthmatic phenotypes for type 2 immune drug assessment; additional IL-5 expression further promotes airway eosinophil infiltration [142]. HDM and pollen-induced humanized models also recapitulate allergen-specific immune responses, facilitating novel immunotherapeutic development [143].

### 5.5. Systemic Sclerosis (SSc) Models

SSc involves widespread tissue fibrosis and vascular injury [144]. PBMC-transplanted NSG mice with bleomycin induction simultaneously reproduce autoimmune reactions, fibrosis, and vascular lesions for drug screening [145]. Another model based on *Rag2*^−/−^*IL2rg*^−/−^ mice displays prominent B-cell infiltration and autoantibody elevation, suitable for anti-CD20 drug evaluation, but cannot simulate skin fibrotic lesions [146].

### 5.6. Neurological Autoimmune Diseases

For multiple sclerosis (MS), MOG peptide-induced humanized NSG models reveal that prior EBV infection exacerbates demyelination, supporting drug screening and patient stratification [147]. The first humanized anti-NMDAR encephalitis model in BRGSF mice reproduces blood-brain barrier damage, and IL-1 blockade relieves neurological symptoms, identifying IL-1β as a therapeutic target [148]. Myasthenia gravis models through PBL or thymus transplantation replicate autoimmune neuromuscular dysfunction for antibody and cell therapy research [149].

### 5.7. Cutaneous Autoimmune Diseases

Humanized atopic dermatitis (AD) and psoriasis vulgaris (PV) models constructed in NSG mice maintain disease-specific immune profiles, with Th2 dominance in AD and IL-23/Th17 activation in PV [150,151]. Psoriatic arthritis (PsA) models in NSG-SGM3 mice fully recapitulate cutaneous and articular lesions, proving that patient immunoglobulins and CD8^+^ T cells jointly drive pathogenesis [152].

## 6. Immune-Reconstituted Humanized Mouse Models for Oncology Research

HIS mouse models have emerged as a core frontier platform for preclinical research in tumor immunotherapy research. By faithfully recapitulating the human immune system and native tumor microenvironment, these models effectively resolve the translational limitations of traditional animal models and streamline the transition from basic research to clinical application [2]. PBMC- and HSC-reconstituted humanized mouse strains, such as NCG and NCG-MHC-dKO mice, serve as invaluable tools for assessing the efficacy, elucidating underlying mechanisms, and predicting the safety of immune checkpoint inhibitors, T cell engagers (TCEs), and cell and gene therapies (CGTs). These refined humanized models drive the innovative development of next-generation anti-tumor immunotherapies and substantially accelerate their clinical translation [153].

### 6.1. Application of Humanized Models in Immune Checkpoint Antibody Therapy

Immune checkpoint inhibitors targeting PD-1/PD-L1 and CTLA-4 represent the most extensively investigated category of immunotherapeutic agents in HIS models, given that their therapeutic mechanism is dependent on the activation of human T cells [154]. In huPBMC-NOG models implanted with lung cancer HCC827 cells or other PD-L1-positive tumor cells, the anti-PD-L1 antibody atezolizumab exerts significant tumor growth inhibition across PBMC donors [155]. In huHSC-NCG models engrafted with triple-negative breast cancer MDA-MB-436 cells, monotherapy with the anti-CTLA-4 agent ipilimumab only moderately suppressed tumor progression, whereas its combined administration with pembrolizumab produced synergistic anti-tumor effects [156]. Moreover, myeloid-enriched humanized models, including NCG-M and NSG-SGM3, are widely utilized in assessing immune checkpoint inhibitors. These models faithfully recapitulate the tumor immune microenvironment, particularly the crosstalk among myeloid-derived suppressor cells (MDSCs), tumor-associated macrophages (TAMs), and lymphocytes, thus supporting more clinically predictive assessment of therapeutic responses [157,158,159,160]. Overall, immune system-humanized mice possess prominent clinical predictive value for the mechanistic research and preclinical efficacy assessment of immune checkpoint inhibitors [161].

### 6.2. Application of Humanized Models in T-Cell Engager (TCE) Therapy

T cell engagers redirect endogenous T cells to eliminate tumor cells and demonstrate potent therapeutic efficacy in hematological malignancies as well as certain solid tumors [156]. Humanized mouse models allow the evaluation of T-cell redirection potency, cytokine release profiles, and off-target toxicities. hPBMC-NCG models, particularly the MHC-deficient NCG-MHC-dKO variant, play a pivotal role in TCE assessment. MHC DKO humanized mouse models exhibit controllably delayed GVHD progression and provide a prolonged experimental observation window [23]. In Hu-PBMC-MHC-dKO NSG models bearing colorectal cancer, the anti-EpCAM/CD3 bispecific antibody achieved complete tumor regression without lethal GVHD occurrence within six weeks, accompanied by marked elevation of IL-2 and IFN-γ levels [162]. A novel CD3 × HER2 TCE significantly suppressed tumor growth and increased intratumoral CD8^+^ T cell infiltration in huHSC-NCG models engrafted with HER2-positive gastric cancer [163]. Collectively, this evidence fully validates that human immune-reconstituted mouse models are uniquely irreplaceable for recapitulating TCE-triggered anti-tumor immune responses and verifying their T cell-dependent therapeutic mechanisms in preclinical in vivo research.

### 6.3. Application of Humanized Models in Cell and Gene Therapy (CGT)

Immune humanized reconstitution models serve as indispensable tools for rigorously evaluating the efficacy, safety, and mechanism of action of CGT agents. These models faithfully recapitulate the human immune system and tumor microenvironment in vivo and have been widely adopted in both PBMC- and HSC-reconstituted experimental systems [164]. MSLN-targeted CAR-T cells exert potent anti-tumor activity by blocking the TGF-β/SMAD signaling pathway, alleviating T cell exhaustion, and remodeling the immunosuppressive tumor microenvironment, as validated using the NCG mouse evaluation platform [6]. Amid the rapid advancement of in vivo CAR-T technology, HIS models serve as indispensable tools for the preclinical exploration and efficacy validation of in vivo CAR-T therapeutics. CD19-targeted in vivo CAR-T has exhibited dose-dependent anti-tumor activity in PBMC humanized MHC-I/II-deficient NSG mice bearing Nalm-6 tumors, leading to complete tumor regression and extended survival [165]. Similarly, CD8-targeted LNP-mediated CAR-T therapy achieved rapid B-cell depletion within 24 h at optimal doses in huHSC-NCG mouse models [6].

Myeloid-enriched humanized models such as NSG-SGM3 and NCG-M support robust reconstitution of human myeloid lineage cells and T cells, offering a unique advantage in evaluating the severity of cytokine release syndrome (CRS) in in vivo CAR-T assessment. These models also serve as promising platforms for studying CAR-T generation, vector immunogenicity, and myeloid-mediated suppression of transduction. NSG-SGM3 humanized mice have been reported to sensitively evaluate the severity of CAR-T-induced CRS, characterized by systemic elevation of CRS-associated cytokines (IL-6, IFN-γ, TNF-α, and GM-CSF) and typical physiological manifestations such as hypothermia and weight loss [166]. In an in vivo CAR-T study, huHSC-NSG-SGM3 mice showed lower CAR-T generation efficiency than conventional huHSC-NSG models, attributable to myeloid cell-mediated vector clearance and innate immune activation [167].

## 7. Discussion

The progressive refinement of immunodeficient mouse models—from nude and SCID mice to NOD/SCID and ultimately to NOD/SCID/*Il2rg^null^* strains—has followed a consistent and logical trajectory: the stepwise elimination of lymphoid and innate immune barriers to maximize human cell engraftment. Across all model generations, a clear cross-model general principle emerges: the degree of human immune reconstitution is inversely correlated with residual murine immunity, particularly NK cell activity, antigen presentation, and phagocytic function. Each advance—whether introducing the NOD background, deleting *Il2rg*, or adding human cytokines—has addressed a specific residual murine barrier, yet new limitations consistently arise, revealing an iterative pattern of gain-and-pain in model development [168].

Despite these advances, a central contradiction persists between engraftment efficiency and experimental fidelity. For instance, Hu PBMC models achieve rapid, high-level human T-cell reconstitution but are severely constrained by GVHD within 4–6 weeks, limiting their use to short-term acute studies. MHC-I/II-dKO strains effectively suppress GVHD and extend the experimental window to 8–12 weeks, yet they may partially compromise human T-cell function due to the absence of murine MHC-mediated T-cell education. Conversely, Hu-HSC and Hu-BLT models support multilineage hematopoiesis and long-term stability with low GVHD, but they require months for reconstitution and involve considerable technical complexity. This trade-off—between speed and physiological relevance—remains unresolved and forces investigators to select models based on narrow experimental endpoints rather than holistic immune function [2].

Synthesis of available evidence from multiple independent studies reveals that no single humanized mouse model recapitulates the full spectrum of human immunity. Instead, each model excels in a specific niche. NOD/SCID/*Il2rg^null^*-based strains (NCG, NSG, NOG) provide the highest engraftment efficiency and longest survival, forming the backbone of most HIS studies. Cytokine-humanized derivatives (NCG-M, NSG-SGM3, NOG-EXL, MITRG, and MISTRG) address the long-standing deficiency in myeloid and dendritic cell reconstitution but at the cost of lineage skewing or, in the case of NSG-SGM3, MITRG, and MISTRG, fatal post-engraftment anemia [25,26]. BRGSF supports tissue-resident macrophages, yet they often require exogenous factors (e.g., FLT3L) to achieve adequate dendritic cell development [43]. TSLP-engineered models (NCG-mTslp, BRGST) uniquely restore secondary lymphoid organogenesis—a critical feature absent in all *Il2rg^null^* models—enabling germinal center responses and affinity-matured antibody production, which are essential for vaccine evaluation [33]. The NCG-Gfi1-KO model introduces an entirely different paradigm: instead of adding human cytokines, it ablates a master regulator of murine granulopoiesis to create a vacant niche for human neutrophil development [78]. Collectively, these models demonstrate that human immune reconstitution can be augmented either by provision of human-specific survival signals or by ablation of murine competitive pathways.

From the perspective of research value and pain points, these models have already demonstrated significant translational utility. They have been successfully applied to evaluate immune checkpoint inhibitors, TCE, and CAR-T therapies, with outcomes showing correlation with clinical data. Myeloid-enhanced models (NSG-SGM3, NCG-M) have proven essential for studying CRS associated with CAR-T therapy, a toxicity poorly modeled in conventional HIS mice [166,167]. However, the pain points are equally evident: the vast diversity of available models—each with unique genetic modifications, cytokine expression patterns, and reconstitution kinetics—creates a lack of standardization across laboratories. This heterogeneity complicates cross-study comparison and hampers the establishment of benchmark performance metrics. Furthermore, most advanced models (BLT) require human fetal tissues, complex surgical procedures, or specialized breeding, making them inaccessible to many academic and industrial laboratories.

While several translational bottlenecks remain that limit the predictive power of these models for clinical outcomes. First, the incomplete recapitulation of human stromal and endothelial niches means that tumor stromal compartments in HIS mice are still largely murine, potentially altering drug distribution, immune cell trafficking, and cytokine signaling. Multiple strategies have been developed to construct humanized stromal microenvironments, including the co-transplantation of mesenchymal stromal cells (MSCs) and HSCs [169,170], co-engraftment of MSCs with endothelial cells or vascular organoids [171,172], humanized ossicle models [173], the human lung-implanted BLT-L models [174], genetically modified MSC-based stromal cell scaffolds [175], and tumor tissue “deconstruction-reconstruction” strategies [121,176,177]. While these approaches have been validated to successfully generate localized humanized stroma, they are inherently limited by regional humanization restricted to graft sites and cannot achieve systemic stromal humanization [107,178,179]. Second, residual mouse innate immunity—particularly macrophages, complement, and some granulocyte populations—can still recognize and respond to human antibodies or engineered T cells, confounding efficacy and toxicity readouts. Third, the vast majority of strains lack human HLA diversity, restricting the study of HLA-restricted antigen presentation and naive T-cell priming, which is critical for vaccine development and personalized immunotherapy [91]. Fourth, even the most advanced models do not fully support the development of human mucosal immunity or tertiary lymphoid structures, limiting their use in studies of chronic infection or autoimmune tissue damage. Addressing these bottlenecks will likely require combinatorial engineering—integrating multiple human transgenes (e.g., HLA class I/II, cytokines, SIRPα, and stromal factors) using precise knock-in technologies such as CRISPR—to create truly humanized immune microenvironments. Until then, the field must accept that current models are complementary rather than universal, and rigorous validation against human clinical data remains essential for each specific application.

## 8. Conclusions

The progressive evolution of immunodeficient mouse models—from early nude mice to advanced NOD/SCID/*Il2rg^null^* strains—has established a robust and versatile foundation for HIS research. Subsequent genetic and physiological refinements have markedly enhanced their translational relevance. These include MHC class I/II ablation to mitigate GVHD, the introduction of human cytokine transgenes to support myeloid and dendritic cell reconstitution, Tslp expression to restore secondary lymphoid organ architecture, and *Gfi1* deficiency to enable functional neutrophil engraftment. Collectively, these innovations have substantially improved immune reconstitution fidelity and broadened the applicability of humanized models across diverse biomedical fields.

These platforms now support mechanistic and efficacy studies in human autoimmunity, cancer immunotherapy—including immune checkpoint inhibitors, TCEs, and CAR-T cell therapies—as well as infectious disease modeling and vaccine evaluation within a physiologically relevant in vivo context. Importantly, they provide critical opportunities to interrogate human-specific immune responses that are not adequately captured by conventional murine systems.

Despite these advances, no single model fully recapitulates the complexity and functionality of the human immune system. Model selection therefore remains highly context-dependent and requires careful consideration of key parameters, including engraftment efficiency, lineage diversity, immune maturation, experimental duration, and cost.

Looking forward, next-generation humanized mouse models are expected to incorporate increasingly sophisticated features, such as HLA matching, human stromal and tissue-specific niches, and finely tuned cytokine expression systems. The integration of CRISPR-based genome engineering and synthetic biology approaches will further enable the development of highly customized, patient-representative “human immune avatars”. Such advances hold significant promise for improving the predictive power of preclinical studies, accelerating translational research, and reducing reliance on non-human primate models.

## Figures and Tables

**Table 1:** Representative NOD/SCID-*Il2rg^null^* and Other High-Immunodeficiency Mouse Strains.

Strain	Full Genetic Design	Construction Strategy	Model Characteristics	Developer/Commercial Source
NSG	NOD.Cg-*Prkdc^scid^ Il2rg^tm1Wjl^*/SzJ	NOD/ShiLtJ genetic background; *Prkdc* mutation; a complete null allele of *Il2rg*	NOD/ShiLtJ genetic background; one of the most severe immunodeficient mouse strains devoid of T, B, and NK cells; highly efficient human cell engraftment; greater radiosensitivity, enabling efficient myeloablation at a relatively low irradiation dose and promoting robust human cell engraftment; in age- and sex-matched cohorts	The Jackson Laboratory (JAX)
NOG	NOD.Cg-*Prkdc^scid^ Il2rg^tm1Sug^*/Jic	NOD/ShiLtJ genetic background; *Prkdc* mutation; truncated *Il2rg* with impaired signaling capacity		Central Institute for Experimental Animals (CIEA)
NCG	NOD/ShiLtJGpt-*Prkdc^em26Cd52^Il2rg^em26Cd22^*/Gpt	NOD/ShiLtJ genetic background; *Prkdc* frameshift mutation; *Il2rg* truncated and signaling-incompetent		GemPharmatech (GPT)
BRGS	BALB/c-*Rag2*^tm1Fwa^ *Il2rg*^tm1Cgn^ *Sirpα*^NOD^	BALB/c genetic background with *Rag2* and *Il2rg* depletion; the endogenous BALB/c *Sirpα* locus was replaced by the NOD-derived *Sirpα* allele	BALB/c genetic background; more radioresistant, making them better suited for the evaluation of Radionuclide Drug Conjugates (RDCs)	Institute Pasteur & genOway

## Data Availability

All raw and processed data generated in this study are available within the manuscript.
